# Epidemiology risk factors and antifungal resistance patterns of *Candida* in cancer patients in Jiangxi China

**DOI:** 10.3389/fmicb.2025.1630226

**Published:** 2025-07-22

**Authors:** Hazrat Bilal, Xiaohui Li, Xunsong Wang, Muhammad Nadeem Khan, Muhammad Shafiq, Jiamei Yu, Hanman Qiu, Qiao-Li Lv, Bin Xu

**Affiliations:** ^1^Jiangxi Key Laboratory of Oncology (2024SSY06041), JXHC Key Laboratory of Tumor Metastasis, NHC Key Laboratory of Personalized Diagnosis and Treatment of Nasopharyngeal Carcinoma, Jiangxi Cancer Hospital & Institute, The Second Affiliated Hospital of Nanchang Medical College, Nanchang, Jiangxi, China; ^2^Department of Medical Laboratory, Jiangxi Cancer Hospital, The Second Affiliated Hospital of Nanchang Medical College, Jiangxi Cancer Institute, Nanchang, Jiangxi, China; ^3^Department of Cell Biology and Genetics, Shantou University Medical College, Shantou, China; ^4^Department of Pharmacology, Research Institute of Clinical Pharmacy, Shantou University Medical College, Shantou, China

**Keywords:** *Candida* species, cancer patients, antifungal resistance, antifungal consumption, epidemiology and risk factors

## Abstract

**Background:**

Candidiasis in cancer patients remains largely unexplored in China. This study examines risk factors and antifungal susceptibility patterns of *Candida* in cancer patients from Jiangxi, China.

**Methods:**

Clinical and demographic data on *Candida* in cancer patients (2018–2024) were retrospectively collected at Jiangxi Cancer Hospital, Nanchang, China. *Candida* distribution across cancers and antifungal susceptibility patterns were analyzed. Risk factors were identified via logistic regression, and antifungal consumption was correlated with *Candida* distribution. Survival probabilities were compared between patients with *C. albicans* and those with non-*albicans Candida* (NAC) infections.

**Results:**

Among 2,761 *Candida* isolates, 1,703 (61.68%) were *C. albicans* and 1,058 (38.31%) were NAC, with a year-wise trend showing a decline in *C. albicans* and a rise in NAC. *C. albicans* was significantly higher in lung (40.57%) and nasopharyngeal (11.33%) cancers, while NAC were more common in gastric (7.56%), colon (8.69%), and urogenital (14.65%) cancers. NAC risk factors included inappropriate empirical therapy (OR 13.8, *P* < 0.001), hypoproteinemia (OR 1.35), anemia (OR 1.28), urinary tract infection (OR 1.71), and indwelling catheters (OR 1.27) (all *P* < 0.05). Radiotherapy, targeted therapy, glucocorticoids, chest tube insertion, and parenteral nutrition were associated with *C. albicans* (*P* ≤ 0.01). Amphotericin B (>99%) and echinocandins (>96%) showed the highest efficacy. *C. tropicalis* displayed notable azole resistance (40.9–74.45%). Caspofungin use negatively correlated with *C. albicans* (*r* = −0.84, *P* = 0.02) and positively with *C. tropicalis* (*r* = 0.78, *P* = 0.04) and *N. glabrata* (*r* = 0.85, *p* = 0.02). NAC infections showed 1.5-fold higher mortality rate than *C. albicans* (95% CI: 1.1–2.0; *P* = 0.0075).

**Conclusion:**

These findings may aid healthcare officials in improving *Candida* management in the region and similar settings.

## Introduction

Cancer patients are highly vulnerable to fungal infections due to immunosuppression from underlying malignancies and treatments (Shelke et al., 2024). *Candida* species are the most commonly reported fungi in cancer patients. They can colonize various body sites, including mucosal membranes, the gastrointestinal tract, and the urogenital tract. This colonization is exacerbated in cancer patients due to immunosuppression, mucosal disruption, and the use of antimicrobials. Colonization, especially at multiple body sites, significantly increases the risk of invasive candidiasis, which may develop within approximately 7 days. Risk factors influencing this transformation include underlying comorbidities, use of indwelling devices, exposure to broad-spectrum antimicrobials, and surgical or invasive procedures (Alenazy et al., 2021; Lass-Flörl et al., 2024). Each year, around 2.5 million people are reported to develop invasive candidiasis, with a mortality rate ranging from 25 to 50% (Bilal et al., 2023).

Historically, *Candida albicans* has been predominantly isolated in cases of candidiasis worldwide. However, recent studies show a shift toward non-*albicans Candida* (NAC) species (Meneghello et al., 2024). This trend is largely attributed to the widespread use of antifungals such as fluconazole and caspofungin. This has led to selective pressure favoring NAC species, which are often less susceptible to azoles and echinocandins.(Lass-Flörl et al., 2024; Lee et al., 2023). The specific distribution of NAC species varies by region and patient population, likely influenced by the use of antifungals and healthcare practices (Bilal et al., 2023; Meneghello et al., 2024; Ngamchokwathana et al., 2021).

In China, antifungal susceptibility patterns vary across regions, and recent studies have noted a decline in susceptibility compared to developed countries (Bilal et al., 2022). Antifungal susceptibility testing often requires a long time. Therefore, guidelines such as those from the European Society of Clinical Microbiology and Infectious Diseases (ESCMID) and the Infectious Diseases Society of America (IDSA) recommend tailoring empirical antifungal therapy based on local species distribution, risk factors, prior antifungal use, and susceptibility data (Pappas et al., 2009; Pappas et al., 2015; Ullmann et al., 2012). In this study, we aimed to analyze the distribution of *Candida* species in various cancer types, along with their associated risk factors and antifungal susceptibility profiles. We also examined the correlation between the consumption of commonly used antifungal drugs and both species distribution and the proportion of non-susceptible isolates in Jiangxi, China, over the past 7 years.

## Materials and methods

### Study setting and design

The current retrospective study was conducted at Jiangxi Cancer Hospital, Nanchang, China. The study center is the only tertiary A-level hospital specializing in cancer diagnosis and treatment in Nanchang, the capital of Jiangxi Province, China. The center is a 2030-bed hospital that provides treatment facilities to over 45 million urban and rural populations. The study was approved by the Ethics and Review Committee of Jiangxi Cancer Hospital (letter number 2024ky078). The committee waived the patient’s consent form due to the study’s retrospective design and the fact that it did not disclose identity of the patients or use their personal information.

### Data collection

This study includes all *Candida* species diagnosed in cancer patients at Jiangxi Cancer Hospital from January 2018 to December 2024. Cases were included if *Candida* species were isolated by the hospital’s clinical microbiology laboratory and if treating physicians had a clinical suspicion of fungal infection based on presenting symptoms and guiedlines established by China Medical Associations (Keighley et al., 2021). For multiple detections of the same species, only the first *Candida* isolate was included in the analysis. The clinical and demographical data related to patient age, gender, Body Mass Index (BMI), Karnofsky Performance Status (KPS), cancer type, cancer treatment, underlying comorbidities, invasive procedures, medical interventions, days stayed in the hospital, 30 days, and all-cause mortality was collected from the hospital’s electronic record. The data on the consumption of antifungal drugs such as fluconazole and caspofungin were collected from the hospital’s information department. Antifungal doses were converted to defined daily doses per 1,000 patients (DDD/1,000 patients) following DDD classification and assignment guidelines (accessed January 12, 2025).^1^ The data related to the antifungal susceptibilities of *Candida* species were collected in the form of MICs detected against each species for each tested drug. Two groups of researchers cross-checked all the data to minimize subjective bias.

### Routine microbiology laboratory protocol

Clinical specimens from suspected infection sites were routinely cultured on Sabouraud dextrose agar and Chromogenic *Candida* medium (Zhengzhou Renfu Bosai Biotechnology Co., Ltd., China). The plates were incubated at 28°C for 24–72 h. A Smart MS 5020 microbial mass spectrometer (Zhuhai Deere Bioengineering Co., Ltd., China) was used for species-level identification, employing matrix-assisted laser desorption ionization time-of-flight mass spectrometry (MALDI-TOF MS). Antifungal susceptibility testing was performed using the YO1O fungal susceptibility panel (Thermo Fisher Scientific, United States). Minimum inhibitory concentrations (MICs) were recorded, and phenotypic resistance or susceptibility was interpreted using CLSI M60 and M57S guidelines (CLSI, 2022; CLSI, 2024). For itraconazole against *C. albicans*, EUCAST breakpoints were used (accessed January 12, 2025).^2^ Where no official breakpoints were available (e.g., 5-flucytosine against all *Candida* species and itraconazole against *P. kudriavzevii*), interpretation was based on published literature (Pfaller et al., 2012).

### Data and statistical analysis

All data were cleaned and processed using Excel 2021. *Candida* isolates were categorized into *C. albicans* and non-*albicans Candida* (NAC) groups. Year-wise incidence and species proportions were analyzed. Chi-square tests were used to compare *C. albicans* and NAC across cancer types, and the distribution of *Candida* species within each cancer type was assessed. Univariate and multivariate logistic regression analyses were conducted to identify risk factors associated with NAC compared to *C. albicans*. Antifungal susceptibility data were analyzed based on MIC values, including MIC ranges, MIC50, MIC90, isolate counts, and the percentages of susceptible/wild-type, resistant/non-wild-type, and intermediate/susceptible dose-dependent isolates for each drug and species. Pearson correlation analysis was performed between the consumption of fluconazole and caspofungin (expressed as DDD/1,000 patient days) and the distribution and proportion of non-susceptible isolates. The survival probabilities of *C. albicans* and the NAC group were evaluated via Kaplan-Meier survival curves analysis. Furthermore, the Cox proportional hazards regression model was used to analyze the risk of death in *C. albicans* versus NAC. All statistical analysis and visualization were performed using GraphPad Prism (v. 9) and R (v. 4.4.1). A *p*-value smaller than 0.05 was considered statistically significant in all cases.

## Results

### Distribution of *Candida* species

**FIGURE 1:**
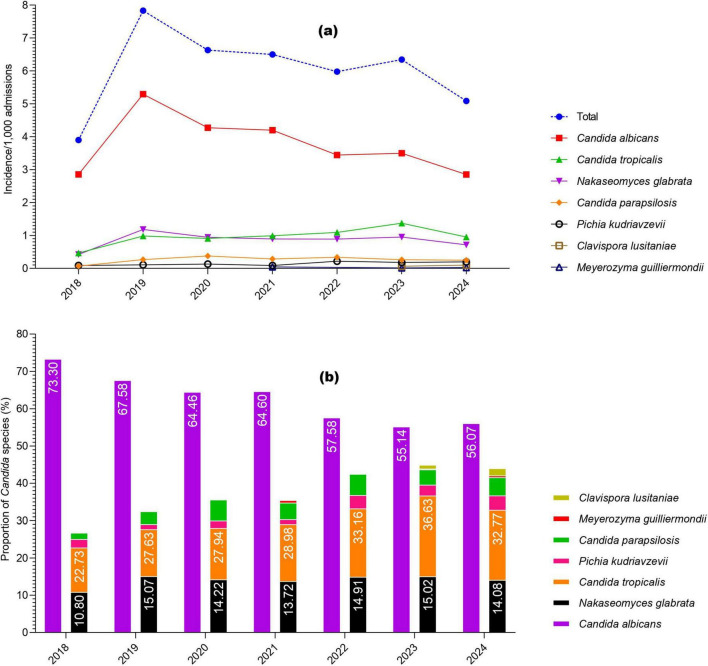
Year-wise occurrence of Candida species in the current study, **(a)** incidence per 1,000 admissions; **(b)** proportions of *Candida* species.

**FIGURE 2:**
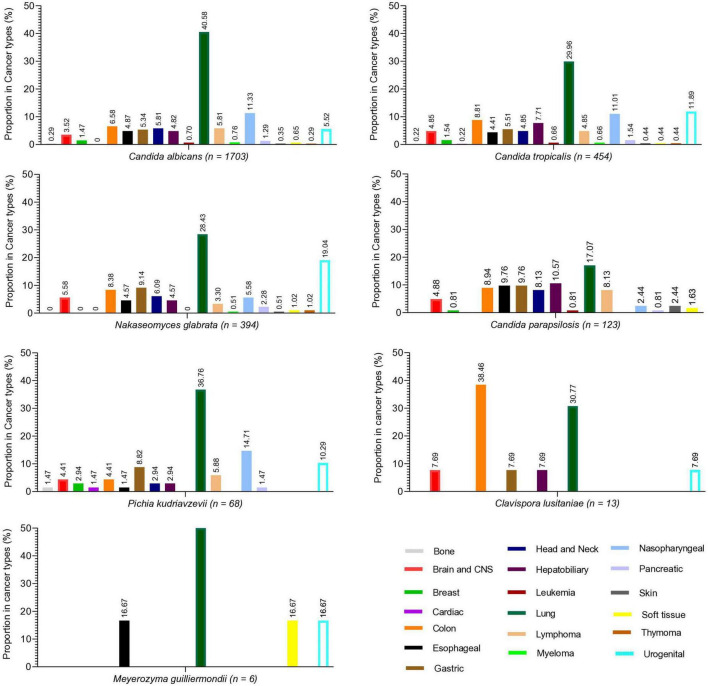
Proportion of different *Candida* species in various cancer types. Each species’ proportion in each cancer type is written at the top of each bar.

**FIGURE 3:**
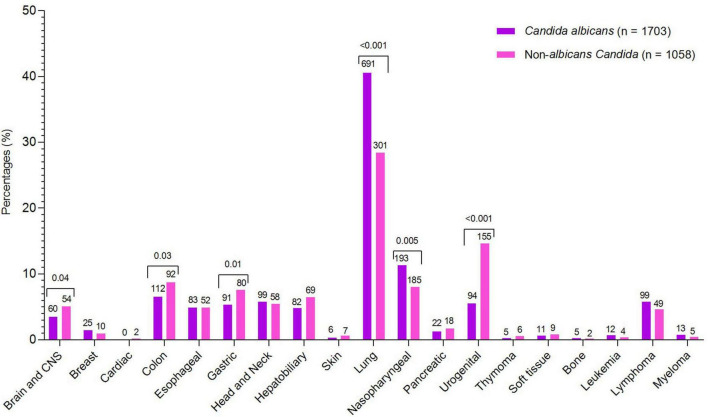
Comparative analysis of *C. albicans* and non-*albicans Candida* across cancer types. Species counts are shown above each bar, with significant *p*-values indicated above the corresponding cancer types.

During the study, a total of 2,761 cases of *Candida* species were reported, of which 1,703 (61.68%) were *C. albicans*, 454 (16.44%) were *C. tropicalis*, 394 (14.27%) were *Nakaseomyces glabrata*, 123 (4.45%) were *C. parapsilosis*, 68 (2.46%) were *Pichia kudriavzevii*, 13 (0.47%) were *Clavispora lusitaniae*, and 6 (0.21%) were *Meyerozyma guilliermondii*. The high incidence of *Candida* species was reported in 2019 (7.89/1,000 admissions), followed by 2020 (6.63/1,000 admissions) and 2021 (6.5/1,000 admissions), while the lowest incidence was observed in 2018 (3.9/1,000 admissions). Overall, *C. albicans* remained more common than NAC, but its proportion declined each year from 2018 to 2024, while NAC rates increased. The year-wise incidence and proportion of *Candida* species are presented in Figure 1. Regarding various cancer types, a high proportion of *Candida* species were reported from lung cancer patients (*n* = 992, 35.93%), followed by nasopharyngeal cancer (*n* = 278. 10.07%), urogenital (*n* = 249, 9.02%), and colon cancer (*n* = 204, 7.38%). A total of 182 (6.59%) cases of hematological cancers were reported, of which 148 were lymphoma, 18 were myeloma, and 16 were leukemia. The proportion of various *Candida* species in different cancer types is presented in Figure 2. Furthermore, the distribution of *C. albicans* versus NAC was analyzed across multiple cancer types (Figure 3). *C. albicans* was significantly more prevalent in lung and nasopharyngeal cancer. In contrast, for the brain and CNS, colon, gastric, and urogenital cancers, the proportions of NAC were significantly higher than those of *C. albicans*.

A large number of cases involved lung cancer patients, leading to most *Candida* isolates being cultured from sputum (71.02%) and throat swabs (4.6%). From blood samples, a total of 39 cases were reported, of which 16 were *C. parapsilosis*, 14 were *C. albicans*, 5 were *C. tropicalis*, 2 were *N. glabrata*, and one each was *P. kudriavzevii* and *M. guilliermondii*. The percentages of *Candida* species reported from various specimen types are shown in Supplementary Figure 1.

### Demographical, clinical, and risk factors analysis of non-*albicans Candida* versus *C. albicans*

The proportions of demographic and clinical variables related to risk factors, along with the univariate regression analysis of NAC versus *C. albicans*, are presented in Table 1. Regarding gender, the proportion of males (*n* = 1876, 67.95%) was higher than females (*n* = 885, 32.05%). The difference between NAC and *C. albicans* regarding gender was statistically insignificant. The median age (IQR) of the NAC group was 65 (55–72), while that for *C. albicans* was 64 (57–71). The median (IQR) for BMI, KPS, and hospital stay duration were slightly higher in the *C. albicans* group compared to the NAC group. Regarding cancer treatments, most patients underwent oncological surgeries (*n* = 844, 30.57%), followed by chemotherapy (*n* = 837, 30.32%), with only 6 patients (0.22%) receiving interventional therapies. The proportion of *C. albicans* was significantly higher in patients who underwent radiotherapy and targeted therapy. Respiratory failure, hypoproteinemia, electrolyte imbalance, anemia, hypokalemia, renal failure, thrombocytopenia, and urinary and biliary tract infections were significantly more common in the NAC group than in the *C. albicans* group. Invasive procedures and medical interventions, such as general surgeries, blood transfusions, mechanical ventilation, indwelling catheters, and inappropriate empirical therapies, were more commonly associated with the NAC group. Conversely, patients receiving glucocorticoids or parenteral nutrition, or those with chest tubes, had a higher proportion of *C. albicans* isolates.

**TABLE 1:** Univariate regression analysis of NAC and *C. albicans.*

Variables	Total = 2,761 (%)	Non-albicans *Candida* = 1,058 (38.31%)	*C. albicans* = 1,703 (61.68%)	OR (95% CI)	*P*
**Demographic and basic characteristics**					
Male	1,876 (67.95)	701 (66.26)	1,175 (69.99)	0.88 (0.75–1.04)	0.14
Female	885 (32.05)	357 (33.74)	528 (31)	1.13 (0.96–1.33)	0.14
Age median (IQR)	65 (56–71)	65 (55–72)	64 (57–71)	0.99 (0.98–1.0)	0.08
BMI median (IQR)	20.87 (18.8–23.44)	20.57 (18.5–23.10)	21.13 (19.03–23.53)	1 (0.99–1)	0.43
KPS median (IQR)	80 (80–90)	80 (70–90)	80 (80–90)	0.98 (0.99–1)	** < 0.001**
Days stay in hospital median (IQR)	22 (14–34)	21 (12–33)	23 (15–35)	0.99 (0.98–1.0)	**0.01**
**Cancer treatment**					
Chemotherapy	837 (30.32)	303 (28.64)	534 (31.36)	0.88 (0.74–1.04)	0.13
Radiotherapy	431 (15.61)	108 (10.21)	323 (18.97)	0.49 (0.38–0.61)	** < 0.001**
Oncologic surgery	844 (30.57)	335 (31.66)	509 (29.89)	1.09 (0.92–1.28)	0.33
Targeted therapy	457 (16.55)	136 (12.85)	321 (18.85)	0.63 (0.51–0.78)	< 0.001
Endocrine therapy	39 (1.41)	16 (1.51)	23 (1.35)	1.12 (0.58–2.12)	0.73
Interventional therapy	6 (0.22)	2 (0.19)	4 (0.23)	0.64 (0.09–2.99)	0.6
Immunotherapy	170 (6.16)	77 (7.28)	93 (5.46)	1.36 (0.99–1.86)	0.05
**Comorbidities and clinical conditions**					
Respiratory failure	316 (11.45)	147 (13.89)	169 (9.92)	1.46 (1.16–1.85)	0.01
Hypoproteinemia	1,006 (36.44)	467 (44.14)	539 (31.65)	1.71 (1.46–2)	< 0.001
Diabetes mellitus	201 (7.28)	70 (6.62)	131 (7.69)	0.85 (0.63–1.14)	0.29
Hypertension	105 (3.8)	32 (3.02)	73 (4.29)	0.7 (0.45–1.05)	0.09
Electrolyte imbalance	342 (12.39)	178 (16.82)	164 (9.63)	1.9 (1.51–2.38)	< 0.001
Cardiovascular disorders	437 (15.83)	182 (17.2)	255 (14.97)	1.18 (0.96–1.45)	0.12
Anemia	710 (25.72)	350 (33.08)	360 (21.14)	1.84 (1.55–2.19)	< 0.001
Myelosuppression	542 (19.63)	190 (17.96)	352 (20.67)	0.84 (0.69–1.02)	0.08
Pneumonia	479 (17.35)	177 (16.73)	302 (17.73)	0.93 (0.76–1.14)	0.5
Hypokalemia	323 (11.7)	142 (13.42)	181 (10.63)	1.3 (1.03–1.65)	0.03
Ascites	129 (4.67)	52 (4.91)	77 (4.52)	1.09 (0.76–1.56)	0.63
Thrombosis	72 (2.61)	33 (3.12)	39 (2.29)	1.37 (0.85–2.2)	0.19
Renal dysfunction/failure	593 (21.48)	266 (25.14)	327 (19.2)	1.41 (1.18–1.7)	< 0.001
Cerebral infarction	41 (1.48)	15 (1.42)	26 (1.53)	0.93 (0.48–1.74)	0.82
Pleural effusion	370 (13.4)	144 (13.61)	226 (13.27)	1.03 (0.82–1.29)	0.8
Hypoalbuminemia	48 (1.74)	13 (1.23)	35 (2.06)	0.59 (0.3–1.1)	0.11
Urinary tract infection	75 (2.72)	42 (3.97)	33 (1.94)	2.09 (1.32–3.34)	< 0.001
Septic shock	101 (3.66)	42 (3.97)	59 (3.46)	1.15 (0.77–1.72)	0.49
Hepatitis B	40 (1.45)	15 (1.42)	25 (1.47)	0.97 (0.5–1.82)	0.91
Abnormal liver function	279 (10.11)	113 (10.68)	166 (9.75)	1.11 (0.86–1.42)	0.43
Thrombocytopenia	158 (5.72)	79 (7.47)	79 (4.64)	1.66 (1.2–2.29)	0.01
Respiratory tract infection	72 (2.61)	25 (2.36)	47 (2.76)	0.85 (0.51–1.38)	0.53
COPD	48 (1.74)	21 (1.98)	27 (1.59)	1.26 (0.7–2.23)	0.44
Biliary tract infection	44 (1.59)	24 (2.27)	20 (1.17)	1.95 (1.07–3.59)	0.03
Neutropenia	202 (7.32)	68 (6.43)	134 (7.87)	0.8 (1.08–0.15)	0.15
**Invasive procedures and medical interventions**					
General surgery	429 (15.54)	184 (17.39)	245 (14.39)	1.25 (1.01–1.54)	0.03
Insulin	531 (19.23)	208 (19.66)	323 (18.97)	1.05 (0.86–1.27)	0.65
Glucocorticoids	2,040 (73.89)	737 (69.66)	1,303 (76.51)	0.71 (0.6–0.85)	< 0.001
Blood transfusion	774 (28.03)	336 (31.76)	438 (25.72)	1.33 (1.13–1.58)	< 0.001
Venipuncture catheterization	1,849 (66.97)	700 (66.16)	1,149 (67.47)	0.95 (0.81–1.12)	0.58
Nasal feeding	276 (10)	114 (10.78)	162 (9.51)	1.13 (0.87–1.45)	0.36
Parenteral nutrition	296 (10.72)	42 (3.97)	254 (14.91)	0.24 (0.17–0.33)	< 0.001
Mechanical ventilation	324 (11.73)	150 (14.18)	174 (10.22)	1.4 (1.11–0.33)	0.01
PICC	383 (13.87)	144 (13.61)	239 (14.03)	0.97 (0.77–1.20)	0.77
CVC	808 (29.26)	315 (29.77)	493 (28.95)	1.04 (0.88–1.23)	0.64
Indwelling catheter	1,270 (46)	538 (50.85)	732 (42.98)	1.37 (1.18–1.6)	< 0.001
Previous antifungal exposure	1,366 (49.47)	521 (49.24%)	845 (49.61)	0.98 (0.84–1.15)	0.87
Chest tube	466 (16.88)	124 (11.72)	342 (20.08)	0.53 (0.43–0.66)	< 0.001
Inappropriate empirical therapy	90/994 (9.05)	76/375 (7.18)	14/619 (2.26)	11.02 (6.32–20.62)	< 0.001
**Lifestyle factors**					
Smoking	814 (29.48)	301 (28.45)	513 (30.12)	0.92 (0.77–1.09)	0.34
Alcohol	335 (12.13)	141 (13.33)	194 (11.39)	1.19 (0.94–1.50)	0.13

Values in bold present significance at < 0.05. OR, odd ratio; CI, confidence interval; IQR, inter quartile range; BMI, body mass index; KPS, karnofsky performance status; COPD, chronic obstructive pulmonary disease; PICC, peripherally inserted central catheter; CVC, central venous catheter.

Furthermore, multivariate regression analysis was performed to determine independent risk factors for NAC and *C. albicans* groups. The inappropriate empirical therapies showed a significantly strong association with the NAC group, with the OR (95% CI) of 13.8 (7.57–26.89) and a *p*-value of < 0.001. Other independent risk factors for NAC included hypoproteinemia (OR = 1.35, *P* = 0.002), anemia (OR = 1.28, *P* = 0.02), urinary tract infection (OR = 1.71, *P* = 0.03), and the use of an indwelling catheter (OR = 1.27, *P* = 0.02). The OR (95% CI) and *p*-value for KPS were 0.99 (0.99–1) and 0.02, respectively, indicating that patients with poor performance status are more likely to develop NAC infections. Conversely, radiotherapy (OR = 0.59, *P* < 0.001), targeted therapy (OR = 0.74, *P* = 0.01), glucocorticoid use (OR = 0.71, *P* < 0.001), chest tube insertion (OR = 0.47, *P* < 0.001), and parenteral nutrition (OR = 0.23, *P* < 0.001) were significantly associated with *C. albicans* group (Table 2).

**TABLE 2:** Multivariate regression analysis of non-*albicans Candida* and *C. albicans* groups.

Variable	OR	95% CI	*P*
(Intercept)	1.31	(0.79 to 2.17)	0.29
**Basic characteristics**			
Days stay in hospital	1	(0.99 to 1)	0.56
KPS	0.99	(0.99 to 1)	**0.02**
**Cancer treatments**			
Radiotherapy	0.59	(0.44 to 0.78)	** < 0.001**
Targeted therapy	0.74	(0.58 to 0.93)	**0.01**
**Comorbidities and clinical conditions**			
Respiratory failure	1.03	(0.76 to 1.38)	0.87
Hypoproteinemia	1.35	(1.11 to 1.65)	**0.002**
Electrolyte imbalance	1.22	(0.94 to 1.59)	0.13
Anemia	1.28	(1.05 to 1.57)	**0.02**
Hypokalemia	1.06	(0.82 to 1.37)	0.65
Renal dysfunction/failure	1.07	(0.87 to 1.31)	0.53
Urinary tract infection	1.71	(1.05 to 2.82)	**0.03**
Thrombocytopenia	1.11	(0.77 to 1.58)	0.58
Biliary tract infection	1.55	(0.82 to 2.95)	0.18
**Invasive procedures and medical interventions**			
General surgery	1.1	(0.88 to 1.38)	0.39
Glucocorticoids	0.71	(0.59 to 0.85)	** < 0.001**
Blood transfusion	1.03	(0.83 to 1.26)	0.81
Parenteral nutrition	0.23	(0.16 to 0.32)	** < 0.001**
Mechanical ventilation	1.29	(0.95 to 1.74)	0.1
Indwelling catheter	1.27	(1.04 to 1.54)	**0.02**
Chest tube	0.47	(0.37 to 0.6)	** < 0.001**
Inappropriate empirical therapy	13.8	(7.57 to 26.89)	** < 0.001**

Values in bold present significance at < 0.05. OR, odd ratio; CI, confidence interval; KPS, karnofsky performance status.

### Antifungal susceptibility profiles

A total of nine antifungal drugs: amphotericin B, anidulafungin, caspofungin, fluconazole, voriconazole, itraconazole, micafungin, posaconazole, and 5-flucytosine, were tested against all *Candida* species. The MIC values analysis and percentage of susceptible/wild types, resistant/non-wild types, and intermediate or susceptible dose-dependent for all species are presented in Table 3. *C. albicans* showed > 99% susceptibility/non-wild types to amphotericin B, echinocandins and azoles such as voriconazole and posaconazole. However, 6.67 and 3.52% of *C. albicans* isolates were non-wild type for itraconazole and 5-flucytosine, respectively. Similarly, 1.23% of *C. albicans* isolates were resistant, and 1.29% were susceptible-dose dependent (SDD) to fluconazole. A high proportion of resistant or non-wild-type *C. tropicalis* isolates was noted against fluconazole, voriconazole, itraconazole, and posaconazole, with percentages of 40.9, 51.32, 74.45, and 64.98%, respectively. However, amphotericin B and echinocandins were effective, with susceptibilities of greater than 99% and greater than 96%, respectively. The resistant proportions of fluconazole, voriconazole, and posaconazole against *N. glabrata* isolates were 7.36, 12.94, and 6.6%, respectively. Amphotericin B (99.49%) remains the most effective drug against *N. glabrata* isolates. Among echinocandins, the lowest susceptibility was reported against micafungin (92.64%), followed by anidulafungin (93.91%) and caspofungin (94.42%). *P. kudriavzevii* isolates were 100% susceptible to amphotericin B and itraconazole. Similarly, 77.94% of *P. kudriavzevii* isolates were susceptible/non-wild types against voriconazole and 5-flucytosine. For *C. parapsilosis*, the lowest susceptibility was reported against fluconazole (79.67%), followed by voriconazole (89.43%). For all other drugs, susceptibility was greater than 90%. Among the 13 tested *C. lusitaniae* isolates, only three were non-wild type against fluconazole, while all remained wild type against the other tested drugs. Similarly, all six *M. guilliermondii* isolates were susceptible to all tested drugs. Amphotericin B was the more effective drug against all species, and echinocandins maintained robust activities.

**TABLE 3:** Antifungal susceptibility profile of *Candida* species in the current study.

Species	MIC																Range	MIC50	MIC90	GM	WT/S (%)	NWT/R (%)	I/SDD (%)
Antifungal drugs	0.0075	0.015	0.03	0.06	0.12	0.25	0.5	1	2	4	8	16	32	64	128	256							
***C. albicans***																							
Amphotericin B					47	721	862	63	7	3							0.12–4	0.5	0.5	0.37	1,700 (99.82)	3 (0.18)	
Anidulafungin		399	449	593	245	10	3	1	1		2						0.015–8	0.06	0.12	0.04	1,698 (99.71)	2 (0.12)	3 (0.18)
Caspofungin		111	918	574	72	24	1	1			2						0.015–8	0.03	0.06	0.04	1,700 (99.82)	3 (0.18)	
Fluconazole					105	704	701	114	32	24	9	8	5		1		0.12–128	0.5	1	0.4	1,660 (97.48)	21 (1.23)	22 (1.29)
Voriconazole	96	1,468	50	34	25	17	11		1	1							0.0075–4	0.015	0.015	0.02	1,678 (98.53)	1 (0.06)	24 (1.41)
Itraconazole		381	610	596	73	24	15	4									0.015–1	0.03	0.06	0.04	1,589 (93.31)	114 (6.69)	
Micafungin	76	1,502	89	23	2	4	3				4						0.0075–8	0.015	0.015	0.02	1,696 (99.59)	4 (0.23)	3 (0.18)
Posaconazole	9	887	661	84	21	10	14	12	2	1	2						0.0075–8	0.015	0.03	0.02	1,642 (96.42)	61 (3.58)	
5-Flucytosine				1,284	292	47	26	6	2	4	2	1		39			0.06–64	0.06	0.12	0.09	1,643 (96.48)	60 (3.52)	
***C. tropicalis***																							
Amphotericin B					9	33	198	200	13		1						0.12–8	0.5	1	0.7	453 (99.78)	1 (0.22)	
Anidulafungin		16	19	67	276	59	6	4			1	6					0.015–16	0.12	0.25	0.12	439 (96.7)	10 (2.2)	5 (1.1)
Caspofungin		17	104	138	107	71	3	4	1	2	2	5					0.015–16	0.06	0.25	0.08	437 (96.26)	14 (3.08)	3 (0.66)
Fluconazole						4	10	56	142	58	14	15	11	11	12	121	0.25–256	4	256	10.11	215 (47.36)	182 (40.09)	57 (12.56)
Voriconazole		11	15	61	141	51	17	13	11	13	121						0.015–8	0.12	8	0.44	233 (51.32)	157 (34.58)	64 (14.1)
Itraconazole		3	6	9	98	168	84	62	6		2	4	12				0.015–32	0.25	1	0.33	116 (25.55)	338 (74.45)	
Micafungin	1	46	293	88	12	2	1	3	2		1	5					0.0075–16	0.03	0.06	0.04	442 (97.36)	11 (2.42)	1 (0.22)
Posaconazole		8	9	45	103	109	75	81	10		5	9					0.015–16	0.25	1	0.28	159 (35.02)	295 (64.98)	
5-Flucytosine				360	51	15	6	7	3	2	2			2	6		0.06–128	0.06	0.12	0.09	414 (91.19)	40 (8.81)	
***Nakaseomyces glabrata***																							
Amphotericin B					30	112	172	66	12	2							0.12–4	0.5	1	0.44	392 (99.49)	2 (0.51)	
Anidulafungin		95	139	70	63	16	3	6	2								0.015–2	0.03	0.12	0.04	370 (93.91)	9 (2.28)	15 (3.81)
Caspofungin		30	144	141	54	19	5	1									0.015–1	0.06	0.12	0.05	372 (94.42)	6 (1.52)	16 (4.06)
Fluconazole					5	3	15	53	95	84	58	28	24	19	2	8	0.12–256	4	32	4.27		29 (7.36)	365 (92.64)
Voriconazole	1	27	44	97	93	79	25	13	8	3	3	1					0.0075–16	0.12	0.5	0.12	343 (87.06)	51 (12.94)	
Itraconazole		14	11	33	97	122	84	26	4				3				0.015–32	0.25	0.5	0.21	391 (99.24)	3 (0.76)	
Micafungin	2	293	48	22	13	6	9	1									0.0075–1	0.015	0.06	0.02	365 (92.64)	15 (3.81)	14 (3.55)
Posaconazole		13	23	29	59	79	87	77	23	1	1	2					0.015–16	0.25	1	0.29	368 (93.4)	26 (6.6)	
5-Flucytosine				348	15	7	5		2	5	10	1			1		0.06–128	0.06	0.12	0.08	376 (95.43)	18 (4.57)	
***Pichia kudriavzevii***																							
Amphotericin B					4	15	31	17	1								0.12–2	0.5	1	0.48	68 (100)		
Anidulafungin		6	17	24	16	3	1	1									0.015–1	0.06	0.12	0.06	66 (97.06)	1 (1.47)	1 (1.47)
Caspofungin		1	1	5	31	22	5	3									0.015–1	0.12	0.5	0.17	60 (88.24)	3 (4.41)	5 (7.35)
Voriconazole		1	2	2	5	32	21	2	3								0.015–2	0.25	0.5	0.29	53 (77.94)	4 (5.88)	11 (16.18)
Itraconazole		2	1	5	12	33	14	1									0.015–1	0.25	0.5	0.21	68 (100)		
Micafungin		7	1	11	42	5		2									0.015–1	0.12	0.25	0.1	66 (97.06)	2 (2.94)	
Posaconazole		2	4	2	6	37	14	2	1								0.015–2	0.25	0.5	0.23	65 (95.59)	3 (4.41)	
5-Flucytosine				5	1		2		6	6	33	14			1		0.06–128	8	16	4.84	53 (77.94)	15 (22.06)	
***Candida parapsilosis***																							
Amphotericin B					15	57	41	8	1		1						0.12–8	0.25	0.5	0.33	121 (98.37)	2 (1.63)	
Anidulafungin		9	8	9	16	9	20	39	12	1							0.015–4	0.5	2	0.32	122 (99.19)		1 (0.81)
Caspofungin		4	14	17	10	29	35	11		1	1	1					0.015–16	0.25	1	0.21	120 (97.56)	2 (1.63)	1 (0.81)
Fluconazole		3				13	44	28	10	10	4	3	5	2		1	0.015–256	1	8	1.06	98 (79.67)	15 (12.2)	10 (8.13)
Voriconazole		80	14	12	4	4	6	2				1					0.015–16	0.015	0.25	0.03	110 (89.43)	3 (2.44)	10 (8.13)
Itraconazole		32	21	30	27	8	3	2									0.015–1	0.06	0.25	0.05	111 (90.24)	12 (9.76)	
Micafungin	1	18	6	5	3	15	21	44	10								0.0075–2	0.5	1	0.29	123 (100)		
Posaconazole		42	42	16	7	7	6	2	1								0.015–256	0.03	0.25	0.04	115 (93.5)	8 (6.5)	
5-Flucytosine				97	12	6	1	2	1		3			1			0.06–64	0.06	0.25	0.09	118 (95.93)	5 (4.07)	

MIC, minimum inhibitory concentration; GM, geometric mean; R, resistant; WT, wild type; S, susceptible; NWT, non-wild type; I, intermediate; SDD, susceptible dose-dependent.

### Correlation analysis of antifungal consumption and *Candida* species distribution and resistance

The data on antifungal consumption for fluconazole and caspofungin were converted to DDD/1,000 patients. For fluconazole, the highest DDDs per 1,000 patient-days were reported in 2020 (5.83), followed by 2023 (5.68) and 2022 (5.48). For caspofungin, the highest value was observed in 2023 (0.69), followed by 2024 (0.59) and 2021 (0.57). The linear regression analysis of fluconazole and caspofungin are shown in Supplementary Figure 2.

A Pearson correlation analysis was performed to examine the relationship between the consumption of caspofungin and fluconazole, species distribution, and the proportion of non-susceptible isolates (Table 4). No significant associations were observed between drug consumption and non-susceptibility, except for N. glabrata, where fluconazole consumption was negatively correlated with the proportion of non-susceptible isolates.

**TABLE 4:** Correlation analysis of caspofungin and fluconazole consumption and distribution and resistant proportion in the current study.

Antifungal drugs/correlation analysis		*Candida albicans*	*Nakaseomyces glabrata*	*Candida parapsilosis*	*Candida tropicalis*	*Pichia kudriavzevii*
**Caspofungin**						
Distribution	*r* (95% CI)	−0.84 (−0.98 to −0.23)	0.85 (0.26 to 0.98)	0.55 (−0.34 to 0.92)	0.78 (0.07 to 0.97)	0.28 (−0.60 to 0.85)
	R squared	0.7	0.72	0.31	0.61	0.08
	*p*-value	**0.02**	**0.02**	0.2	**0.04**	0.54
Non-susceptible	*r* (95% CI)	0.54 (−0.36 to 0.92)	−0.6 (−0.93 to 0.27)	−0.23 (−0.84 to 0.63)	0.28 (−0.60 to 0.85)	0 (−0.75 to 0.75)
	R squared	0.29	0.36	0.05	0.08	0
	*p*-value	0.21	0.15	0.62	0.54	0.99
**Fluconazole**						
Distribution	r (95% CI)	−0.63 (−0.94 to 0.23)	0.57 (−0.31 to 0.93)	0.78 (0.07 to 0.97)	0.58 (−0.31 to 0.93)	0.24 (−0.63 to 0.84)
	R squared	0.4	0.33	0.61	0.33	0.06
	*p*-value	0.13	0.18	**0.04**	0.18	0.6
Non-suspectable	*r* (95% CI)	0.12 (−0.70 to 0.80)	−0.8 (−0.97 to −0.11)	−0.3 (−0.86 to 0.58)	−0.2 (−0.83 to 0.65)	-
	R squared	0.01	0.64	0.09	0.04	-
	*p*-value	0.8	0.03	0.51	0.66	-

Values in bold present significance at < 0.05.

Notably, caspofungin consumption showed a significant negative correlation with C. albicans distribution (*r* = −0.84, *P* = 0.02) and significant positive correlation with C. tropicalis (*r* = 0.78, *P* = 0.04) and N. glabrata (*r* = 0.85, *P* = 0.02), suggesting a shift in Candida distribution from C. albicans to NAC species. Similarly, fluconazole consumption was significantly positively correlated with C. parapsilosis distribution (*r* = 0.78, *P* = 0.04), indicating a possible selective pressure favoring the proliferation of NAC species.

### Survival analysis

**FIGURE 4:**
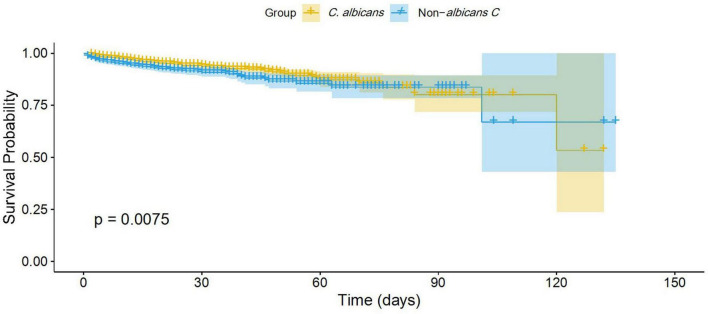
Kaplan-Meier survival curve analysis of *C. albicans* versus non-*albicans Candida* group.

A total of 185 (6.7%) 30-day mortality cases and 211 (7.64%) all-cause mortality cases were reported across all *Candida* cases. For the NAC group, the 30-day and all-cause mortality rates were 90 (8.69%) and 102 (9.85%), respectively. For the *C. albicans* group, these rates were 95 (5.58%) and 109 (6.4%), respectively. The Kaplan-Meier survival analysis revealed that patients infected with NAC species had a significantly lower survival probability than those infected with *C. albicans* (*p* = 0.0075, log-rank test) (Figure 4).

**FIGURE 5:**
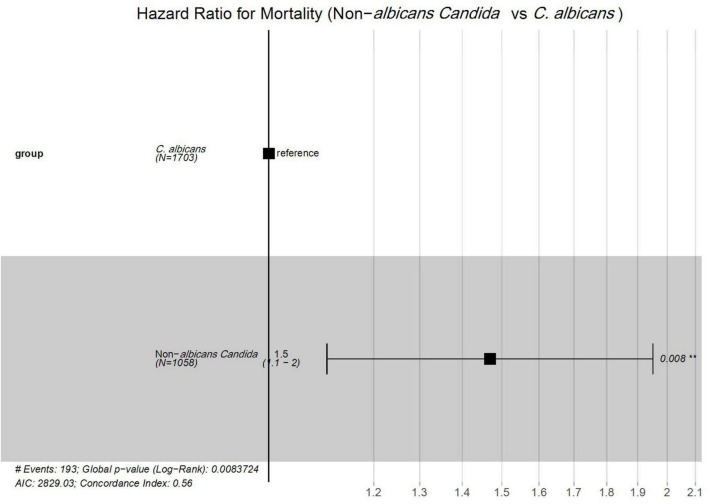
Cox proportional hazards analysis of mortality risk between *Candida albicans* and non-*albicans Candida* groups. #; Numbers, **; Significant.

Furthermore, the Cox proportional hazard model was applied to determine the mortality risk for NAC versus *C. albicans* groups. The result revealed that NAC cases had a 1.5-fold higher risk of mortality compared to *C. albicans* cases (HR = 1.5; 95% CI: 1.1–2.0; *P* = 0.008) (Figure 5).

## Discussion

*Candida* infection is common in cancer patients due to their immunocompromised status, requiring timely management. However, studies focusing on *Candida* isolates from cancer patients in China are limited (Sharifi et al., 2023). This study comprehensively analyzed the species distribution, risk factors, and antifungal susceptibilities of *Candida* isolates from cancer patients in Jiangxi, China. Overall, *C. albicans* remains dominant in our study; however, the year-wise trend showed a decreasing incidence of *C. albicans* and an increasing trend of NAC. This shifting epidemiological landscape aligns with the previously published literature from China (Khan et al., 2025). Among the NAC group, *C. tropicalis* was the most frequently reported, followed by *N. glabrata* and *C. parapsilosis*. The high proportion of *C. tropicalis* in our study aligns with previous findings, contrasting with Europe and the United States, where *N. glabrata* is more common (Ricotta et al., 2020; Wang et al., 2021; Wolfgruber et al., 2024). The variation in species distribution might be due to differences in healthcare practice, patients’ demographics, and regional latitudes (Bilal et al., 2022; Khan et al., 2025). Among the sample types, sputum and throat swabs were the most frequent in our study. This is due to the high proportion of lung and nasopharyngeal cancer cases, for which sputum and throat swab collection is a less invasive and more patient-friendly method to obtain biological specimens (Lucaciu et al., 2024; Sharifi et al., 2023). *C. parapsilosis* was predominantly found in blood samples, consistent with its known role in candidemia, due to its affinity for intravascular catheters and ability to form biofilms (Franconi et al., 2023).

Among the cancer types, *Candida* species were most commonly isolated from patients with lung and nasopharyngeal malignancies. This may be due to their anatomical site, as the respiratory and upper aerodigestive tracts are exposed to the external environment, which poses a high risk of colonization and infection (Liu et al., 2025). Furthermore, radiotherapies in these cancers can cause salivary gland dysfunction, mucositis, and alterations in oral flora, which facilitate *Candida* colonization and infection (Bhumitrakul et al., 2024). NAC was significantly predominant in gastric, colon, and urogenital cancers, while *C. albicans* was significantly more prevalent in lung and nasopharyngeal cancers. The unique mucosal environment and frequent exposure to broad-spectrum antimicrobials might selectively favor the colonization of NAC in gastrointestinal and urogenital cancer (Sun et al., 2025). Conversely, the commensal nature of *C. albicans* in the oropharyngeal tract and its adaptability to an oxygen-rich environment might promote its colonization in infection among lung and nasopharyngeal cancer patients (Debta et al., 2022).

Regarding risk factor analysis, inappropriate empirical therapies were significantly associated with the NAC group. This may be due to the lower susceptibility of NAC species to commonly used empirical treatments, leading to delayed infection clearance and enabling their proliferation (Katsipoulaki et al., 2024). Furthermore, correlation analysis was performed with caspofungin and fluconazole consumption, with the distribution of *Candida* species, and the proportion of non-susceptibility. Notably, a negative correlation was observed between caspofungin consumption and *C. albicans* distribution, and positive correlations were observed with *N. glabrata* and *C. tropicalis*. Similarly, a positive correlation was found between fluconazole consumption and *C. parapsilosis*. This finding reinforces our previous observation of the epidemiological shift of *C. albicans* to NAC, as well as the significant association between inappropriate therapies and the NAC group. The *C. albicans* are comparatively more susceptible to antifungal drugs and are mostly cleared with empirical treatments. This selective pressure promotes the emergence of less susceptible NAC species (Dawoud et al., 2024). Besides this, hypoproteinemia, anemia, UTI, and indwelling catheter use were observed as independent risk factors for the NAC group. The low serum protein level and anemia indicate impaired immune function and oxygen delivery, favoring the proliferation of NAC species (Jørgensen, 2024). Similarly, UTI disrupts the normal urogenital flora, providing a conducive environment for NAC species colonization and infection. Catheterization is a well-documented risk factor for NAC, as *C. parapsilosis* and *C. tropicalis* form biofilms on its surface, facilitating candidiasis (Wijaya et al., 2023; Yamin et al., 2021). Radiotherapy, targeted therapy, glucocorticoid use, chest tube insertion, and parenteral nutrition were independent risk factors for the *C. albicans* group. Studies have shown that radiotherapy and glucocorticoid use impair mucosal immunity, promoting the adhesion and invasiveness of *C. albicans* (Pasman et al., 2022). Targeted therapy contributes to immunosuppression, promoting the pathogenic transformation of commensal *C. albicans* (Feng et al., 2024). Similarly, chest tubes and parenteral nutrition provide direct access to a nutritionally rich environment for the proliferation of *C. albicans* species (Cinti et al., 2024; Nagar et al., 2023).

Regarding the antifungal susceptibility profiles, amphotericin B was the most susceptible drug against all *Candida* species, reaffirming its status as a potent broad-spectrum antifungal agent. *C. albicans* isolates were susceptible primarily to the tested antifungal agents, except for itraconazole and 5-flucytosine, for which 6.67 and 3.52% of the tested isolates were non-wild types. These findings align with prior studies, which report generally high but not absolute susceptibility to tested antifungal agents (Bilal et al., 2022). The occasional resistance may be linked to mutations in genes involved in ergosterol biosynthesis, efflux pump regulation, cytosine permease function, and deaminase activity (Huang et al., 2025). Among the NAC, the azole drugs were less susceptible compared to other antifungal agents. Specifically, the *C. tropicalis* isolates showed high resistance to all azole drugs, with rates of 40.9, 51.32, 74.45, and 64.98% against fluconazole, voriconazole, itraconazole, and posaconazole, respectively. A previously published systematic analysis of data from the past 10 years in China reported high resistance rates, with 22.05% of C. tropicalis isolates being non-susceptible to fluconazole and 16.9% to itraconazole (Bilal et al., 2022). This high resistance to azole drugs reflects emerging regional resistant trends due to its widespread use. Previous literature attributes such resistance to mechanisms such as ERG11 mutation and efflux pump overexpression (Siqueira et al., 2025). The echinocandins showed a comparatively less resistant rate (<5%) against all NAC species. However, careful administration of echinocandin drugs is required, as some recent studies have reported the emergence of resistance due to mutations in the hotspot region of the FKS1 and FKS2 genes (ElFeky et al., 2025; Huang et al., 2025). Notably, 22.06% of *P. kudriavzevii* isolates were non-wild-types against 5-flucytosine. The resistance has been linked in previous reports to mutations in cytosine permease (FCY2) and deficient cytosine deaminase activity, which impair 5-flucytosine uptake and metabolism (Czajka et al., 2023). These mutations are reportedly more prevalent in *P. kudriavzevii* than in other NAC species (Jamiu et al., 2020). Therefore, based on the current literature, monotherapy with 5-flucytosine for *P. kudriavzevii* is often unsuitable and may require combination therapy with other antifungal agents such as echinocandins or amphotericin B (Nguyen et al., 2024).

Overall, the mortality rate in our study (6–10%) was comparatively lower than that reported in other studies from China, which have documented rates ranging from 20 to 50% (Bilal et al., 2023; Dai et al., 2025; Zhang et al., 2019). Our lower mortality rate may be attributed to the inclusion of all *Candida* cases, rather than only those involving invasive or bloodstream infections, which are typically associated with a higher mortality rate (Kwon et al., 2021). When comparing survival probabilities between the *C. albicans* and NAC groups, the NAC group showed a lower survival probability and a 1.5-fold higher mortality risk. The finding of a high mortality risk in the NAC group aligns with previous results reported by other researchers. This higher mortality risk associated with NAC may be attributed to their antifungal resistance mechanisms, particularly against azole drugs, and delays in initiating appropriate empirical therapies (Chastain et al., 2024; Hoenigl et al., 2024).

The increasing prevalence of azole-resistant non-albicans *Candida* species in our oncology patients highlights the need to reconsider empirical antifungal therapy, favoring agents such as amphotericin B and echinocandins, which have shown higher susceptibility. This aligns with IDSA guidelines, which recommend echinocandins as first-line treatment in high-resistance settings (Pappas et al., 2015). Incorporating local resistance data into antifungal stewardship programs can promote targeted, timely therapy, reduce unnecessary azole use, and prevent the development of further resistance. Stewardship efforts, including ongoing surveillance, clinician education, and region-specific treatment protocols, are crucial in oncology care to optimize antifungal use, enhance patient outcomes, and preserve drug efficacy (Fisher et al., 2022).

The limitations of the present study include its retrospective nature, single-center design, and the unavailability of isolates for molecular analysis, such as genotyping and detection of resistance mechanisms, including ERG11 and FKS mutations. Additionally, clearly distinguishing between colonization and invasive candidiasis was impossible due to the absence of some clinical details, such as radiological and biomarker data. These factors may limit the external validity of the findings, though they remain clinically relevant within similar hospital settings. Nevertheless, we included all culture-positive cases in which physicians had a clinical suspicion of fungal infection, reflecting real-world diagnostic practices. Despite these limitations, our study remains distinctive in providing a thorough evaluation of *Candida* cases among cancer patients, a dataset that is rarely available. The findings from this work are expected to aid healthcare professionals in more effectively managing *Candida* infections in similar clinical settings.

## Conclusion

This study retrospectively analyzed the species distribution, risk factors, and antifungal susceptibility patterns of *Candida* isolates among cancer patients in Jiangxi, China. While *C. albicans* remained the predominant species, a year-wise trend indicated a declining proportion of *C. albicans* and a rising incidence of NAC. Caspofungin consumption showed a negative correlation with *C. albicans* and positive correlations with *N. glabrata* and *C. tropicalis* distribution. A higher proportion of *C. albicans* isolates were recovered from lung and nasopharyngeal cancers, whereas NAC species were more frequently associated with gastric, colon, and urogenital cancers. Inappropriate empirical therapy, hypoproteinemia, anemia, and the use of indwelling catheters were identified as independent risk factors for NAC infections. In contrast, radiotherapy, targeted therapy, and glucocorticoid use were significantly associated with *C. albicans*. The NAC group exhibited a 1.5-fold higher mortality risk than the *C. albicans* group. Amphotericin B and echinocandins demonstrated good activity against Candida species, whereas lower susceptibility rates to azole drugs were observed, particularly among NAC isolates. Future large-scale, multicenter molecular studies are warranted to investigate emerging azole resistance mechanisms in NAC species.

## Data Availability

The raw data supporting the conclusions of this article will be made available by the authors, without undue reservation.

## Footnotes

1
https://atcddd.fhi.no/

2
https://www.eucast.org/astoffungi/clinicalbreakpointsforantifungals
