# Supplementary material for: Epidemiology risk factors and antifungal resistance patterns of Candida in cancer patients in Jiangxi China
Source: Front Microbiol. 2025 Jul 22;16:1630226. doi: 10.3389/fmicb.2025.1630226 (PMC12321901; doi:10.3389/fmicb.2025.1630226)
Supplement: Supplementary file 1 [file Data_Sheet_1.pdf]

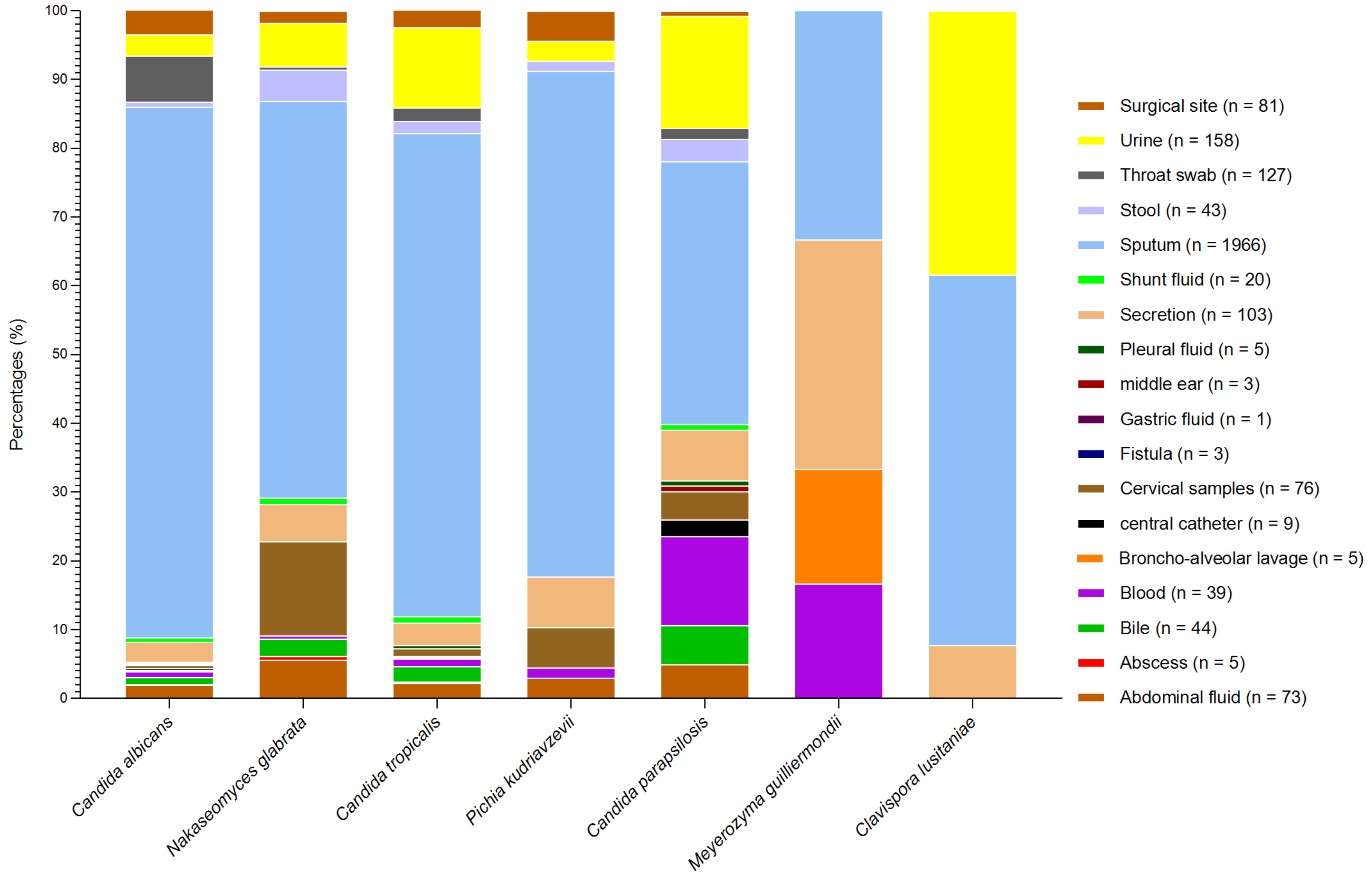

Figure S1: percentages of *Candida* species from various specimen types.

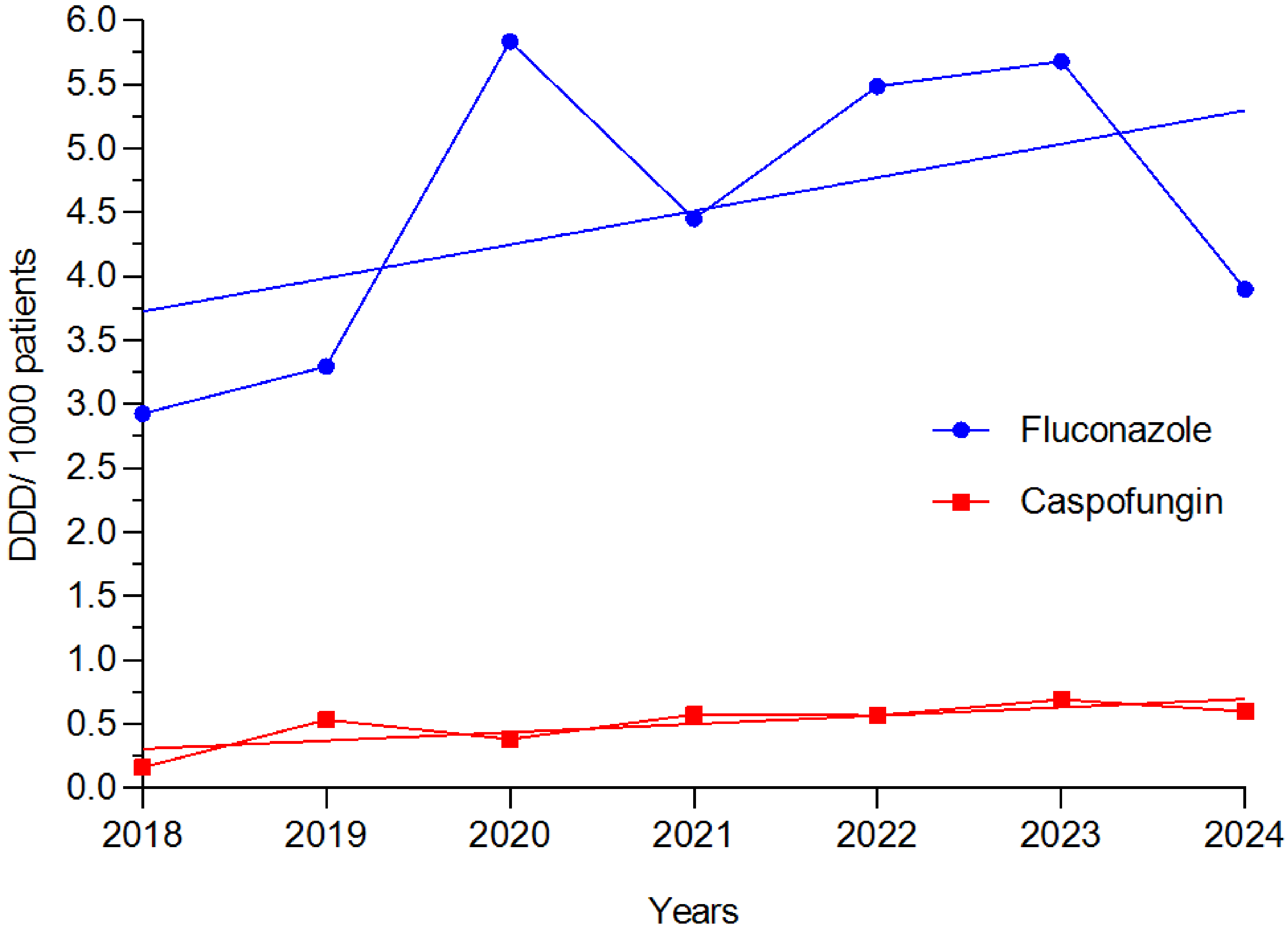

Figure S2: Linear regression analysis of fluconazole and caspofungin.
